# MicroRNA-18a-5p Suppresses Tumor Growth *via* Targeting Matrix Metalloproteinase-3 in Cisplatin-Resistant Ovarian Cancer

**DOI:** 10.3389/fonc.2020.602670

**Published:** 2020-12-17

**Authors:** Blanca I. Quiñones-Díaz, Jeyshka M. Reyes-González, Victoria Sánchez-Guzmán, Isabel Conde-Del Moral, Fatma Valiyeva, Ginette S. Santiago-Sánchez, Pablo E. Vivas-Mejía

**Affiliations:** ^1^ Department of Biochemistry, University of Puerto Rico, San Juan, Puerto Rico; ^2^ Department of Interdisciplinary Sciences, University of Puerto Rico, San Juan, Puerto Rico; ^3^ Department of Chemistry, University of Puerto Rico, San Juan, Puerto Rico; ^4^ Comprehensive Cancer Center, University of Puerto Rico, San Juan, Puerto Rico

**Keywords:** ovarian cancer, cisplatin resistance, miR-18a, MMP-3, folate-liposomes

## Abstract

Cumulating evidence indicates that dysregulation of microRNAs (miRNAs) plays a central role in the initiation, progression, and drug resistance of cancer cells. However, the specific miRNAs contributing to drug resistance in ovarian cancer cells have not been fully elucidated. Aimed to identify potential miRNAs involved in platinum resistance, we performed a miRNA expression profile in cisplatin-sensitive and cisplatin-resistant ovarian cancer cells, and we found several differentially abundant miRNAs in the pair of cell lines. Notably, miR-18a-5p (miR-18a), a member of the oncogenic associated miR-17-92 cluster, was decreased in cisplatin-resistant as compared with cisplatin-sensitive cells. Real-time PCR analysis confirmed these findings. We then studied the biological, molecular, and therapeutic consequences of increasing the miR-18a levels with oligonucleotide microRNA mimics (OMM). Compared with a negative control OMM, transient transfection of a miR-18a-OMM reduced cell growth, cell proliferation, and cell invasion. Intraperitoneal injections of miR-18a-OMM-loaded folate-conjugated liposomes significantly reduced the tumor weight and the number of nodules in ovarian cancer-bearing mice when compared with a control-OMM group. Survival analysis using the Kaplan-Meier plotter database showed that ovarian cancer patients with high miR-18a levels live longer in comparison to patients with lower miR-18a levels. Bioinformatic analyses, real-time-PCR, Western blots, and luciferase reporter assays revealed that Matrix Metalloproteinase-3 (MMP-3) is a direct target of miR-18a. Small-interfering RNA (siRNA)-mediated silencing of MMP-3 reduced cell viability, cell growth, and the invasiveness potential of cisplatin-resistant ovarian cancer cells. Our study suggests that targeting miR-18a is a plausible therapeutic strategy for cisplatin-resistant ovarian cancer.

## Introduction

Ovarian cancer is the deadliest gynecological malignancy, provoking around 239,000 new cases and 152,000 deaths worldwide annually (1). The standard treatment for women with ovarian cancer consists of debulking cytoreductive surgery and platinum/taxane combination chemotherapy (2). Despite the initial effectiveness of the combined chemotherapy, more than 70% of ovarian cancer patients relapse and they become resistant to platinum-based treatment (3). Several mechanisms of cisplatin resistance have been proposed, including increased drug efflux, decreased drug internalization, drug inactivation, and impairment of DNA damage repair mechanisms (4–6). Other mechanisms of cisplatin resistance include activation of cell survival pathways, dysregulation of oncogenes, tumor suppressor genes, long-non-coding RNAs and microRNAs (7).

MicroRNAs (miRNAs) are small non-coding RNAs that regulate gene expression at the posttranscriptional level. Evidence indicates that miRNAs potentially regulate more than 60% of the protein-coding genes (8). Mechanistically, miRNAs bind mainly to the 3′ untranslated region (3′UTR) of their target messenger RNAs (mRNAs) and induce mRNA degradation or inhibit translation initiation (9). Dysregulation of miRNAs is a phenomenon commonly observed in most cancer types (10). In cancerous cells, miRNAs that are anomalously upregulated are referred as oncomiRs (11). Conversely, downregulated microRNAs are named tumor-suppressor miRNAs (11).

Increased levels of members of the miR-200 family, miR-199a, miR-21, miR-203, and decreased levels of miR-140, miR-145, and miR-31 have been reported in ovarian cancer cells and human ovarian cancer samples (12–14). Most of these miRNAs play a central role in ovarian cancer initiation, progression, epithelial-to-mesenchymal transition, metastasis, and drug resistance (13–15). Some of them have been proposed as diagnostic and/or prognostic markers, and as targets for ovarian cancer therapy. Nevertheless, the precise miRNAs contributing to the cisplatin resistance of ovarian cancer cells remain partially elusive.

Aimed to identify the key miRNAs associated with the cisplatin resistance of ovarian cancer cells, we performed a miRNA expression array. A list of 26 miRNAs, including various members of the miR-17-92 cluster, were differentially abundant in cisplatin-sensitive and cisplatin-resistant ovarian cancer cells. Although members of this cluster have been reported as oncomiRNAs and are upregulated in several cancers, we observed opposite tendencies, as they were decreased in cisplatin-resistant in comparison with cisplatin-sensitive ovarian cancer cells. We used oligonucleotide microRNA mimics (OMMs) to increase the levels of downregulated miRNAs and noticed that the OMM of miR-18a-5p prominently reduced cell proliferation in a clonogenic assay. Thus, we focused our studies on the biological consequences of increasing the miR-18a levels in ovarian cancer cell lines and a xenograft ovarian cancer mouse model. We conducted a survival analysis using the Kaplan-Meier plotter database, which revealed that miR-18a is a clinically relevant target in ovarian cancer. Then, we performed bioinformatic analyses, real-time PCR, Western blot analysis, and luciferase reporter assays to identify the potential miR-18a target genes. These studies revealed that MMP-3 is a direct target of miR-18a in ovarian cancer cells. Finally, we used siRNAs to silence MMP-3 and observed a significant reduction in cell growth, cell viability, and the invasion ability of cisplatin-resistant ovarian cancer cells. Taken together, this study proposes miR-18a as a promising therapeutic target in cisplatin-resistant ovarian cancer and validates MMP-3 as a direct miR-18a target in cisplatin-resistant ovarian cancer cells.

## Materials and Methods

### Cell Culture

The human epithelial ovarian cancer cells A2780 and A2780CIS were purchased from the European Collection of Cell Cultures (ECACC). A2780CP20 cells were kindly gifted by Dr. Anil K. Sood (MD Anderson Cancer Center, Houston, TX) (16). High-grade serous ovarian cancer (HGSOC) cells OV-90 and OVCAR3 were purchased from ATCC (Chicago, IL). Cisplatin-resistant OV90CIS and OVCAR3CIS were generated by sequential addition of increasing concentrations of cisplatin to the parental cell lines (17). The chemosensitivity of the generated cell lines was assessed by dose-response experiments with cisplatin. The IC50 of this panel of cells has been reported (17). A2780, A2780CP20, and A2780CIS cells were maintained in RPMI-1640 (HyClone, Logan, UT), and OVCAR3 and OVCAR3CIS were maintained in RPMI-1640 supplemented with 0.01 mg/mL insulin (Sigma-Aldrich, St Louis, MO). OV-90 and OV-90CIS cells were cultivated on a 1:1 (v/v) ratio of M199 media and MCDB 105 media. All media was supplemented with 10% FBS and 1% antibiotics. For experiments, all cells were kept at 37°C and 5% CO_2_ atmosphere. Experiments were performed at 60–80% confluency.

### RNA Isolation for miRNA Expression Profiles

Total RNA was isolated from ovarian cancer cells using the mirVana™ miRNA Isolation Kit (Invitrogen, ThermoFisher Scientific, Carlsbad, CA) following the manufacturer’s instructions. Total RNA was eluted with pre-heated water, and RNA concentration was determined using a Thermo Scientific NanoDrop spectrophotometer.

### miRNA Expression Profiling

Total RNA from A2780, A2780CP20, and A2780CIS ovarian cancer cells was biotin-labeled following the FlashTag Biotin HSR RNA labeling kit manufacturer’s instructions (Affymetrix) and subjected to Affymetrix GeneChip miRNA 2.0 Arrays. Array hybridization and analysis were performed by the Genomics Core Facility at Brown University (Providence, RI). MiRNA arrays were performed in duplicate. Differentially expressed miRNAs were chosen using a fold change of at least 1.5 between A2780 vs. A2780CIS, and A2780 vs. A2780CP20 with a P-value of at least 0.05.

### Quantitative Real-Time PCR (qRT-PCR) Analysis for miRNAs

Total RNA (10 ng) was reverse transcribed and subjected to TaqMan miRNA assays (Applied Biosystems, ThermoFisher Scientific, Foster City, CA) in a StepOne plus thermal cycler system (Applied Biosystems). Briefly, total RNA was combined with RT Master Mix and gently-mixed. Next, 5X RT primer for miRNA assay was added into the RT reaction tube, mixed, and incubated on ice for 5 min. RT reaction protocol for thermal cycler was as follows: 30 min at 16°C, 30 min at 42°C, and 5 min at 85°C in a Veriti thermal cycler (Applied Biosystems). For the qPCR analysis, the cDNA was added to the reaction mix containing TaqMan^®^ Universal Master Mix II, with UNG, 20X TaqMan^®^ MicroRNA Assay (primer), and nuclease-free water, and mixed. qPCR reaction conditions were followed as per manufacturer instruction. U44 RNA was used as an internal control. Relative miRNA expression was calculated by the ΔΔCt-method using the StepOne Software version 2.1 from Applied Biosystems.

### *In Vitro* miRNA and siRNA Transfection

One day before transfections, cells (3.0 x10^4^ cells/ml) were plated in 10-cm Petri dishes. Mature miRNA and siRNAs sequences are shown in **Supplementary Table S1**. OMMs or siRNAs (Sigma-Aldrich) (100 nM final concentration) were mixed with HiPerfect (Qiagen, Germantown, MD) on a 1:2 volume ratio (siRNA/OMM: HiPerfect) and incubated for 15–20 min in serum and antibiotic-free Opti-MEM medium at room temperature. The cell culture media of the cells was replaced with Opti-MEM, and the transfection mixture was added dropwise. Transfected cells were incubated overnight, and the next day cell pellets were collected for subsequent experiments.

### Cell Viability, Cell Proliferation, and Invasion Assays

For cell viability assays, cells (3.0 x 10^4^ cells/mL) were seeded on 96-well plates. The next day different concentrations of siRNAs were added to the cells as described above. Twenty-four h after transfection, fresh media was added to the cells. Seventy-two h post-transfection, AlamarBlue (ThermoFisher Scientific) was added, and cells were incubated for 3 h at 37°C. Absorbance values were obtained spectrophotometrically (570 nm) in a plate reader (BioRad). Percentages of cell viability were obtained taking the values of the untreated cells as 100% of viability. Cell growth was assessed with clonogenic assays. Briefly, cells were seeded into 6-well plates, and 24 h later, cells were transfected with 100 nM (final concentration) OMM or siRNAs as described above. The next day, transfected cells (1,000 or 2,500) were seeded in 10-cm Petri dishes. Colonies formed after seven days were stained with 0.5% crystal violet in methanol. Colonies of at least 50 cells were quantified under a light microscope (CKX41; Olympus) at 10X magnification in five random fields. Percentages of clonogenicity were calculated relative to the control (CNT). For short-term cell proliferation assays, the CyQUANT^®^ Direct Cell Proliferation assay kit from Invitrogen was used according to the manufacturer’s instructions. Briefly, A2780CP20 cells (3 x 10^4^ cells/mL) were seeded into 6-well plates. Twenty-four h later, OMMs (50 nM) were added to the cells. Eighteen h after transfection, 5,000 cells were seeded into 96-well plates and incubated at 37°C. After 24, 48, and 72 h, RPMI-1640 medium containing DNA-binding dye and background suppressor was added to the cells and incubated for 1 h at 37°C. Fluorescence intensity at 480 nm excitation and 535 nm emission was measured using a Varioskan Flash reader from Thermo Scientific. To assess cell invasion, cells (2 x 10^4^ cells/mL) were seeded in 10-cm Petri-dishes. Twenty-four hrs later, cells were transfected with OMMs or siRNAs (25 nM final concentration). The next day, 70,000 cells were seeded into matrigel-coated transwells. Forty-eight h later, cells were fixed and stained using the Fisher HealthCare™ PROTOCOL™ Hema 3™ Manual Staining System. The invading cells were counted at 20X on an Olympus 1X71 microscope equipped with a digital camera (Olympus DP26). Percentages of invaded cells were calculated, taking the untransfected cell values as 100% of cell invasion.

### Liposome Preparation, Tumor Implantation, and Mice Treatment

DOPC (1,2-dioleoyl-sn-glycero-3-phosphocholine) and cholesterol were purchased from Avanti Polar Lipids (Alabaster, AL) and DSPE-PEG(2000)-Folic acid (1,2-distearoyl-sn-glycero-3-phosphoethanolamine-N-[folate(polyethylene glycol)-2000]) was purchased from Nanocs (New York, NY). To prepare liposomes, 5 µg of OMMs were mixed with DOPC (1:10 w/w, OMM: DOPC), DSPE-PEG(2000)-Folic Acid (5% mol/mol DOPC) and cholesterol (25% w/w DOPC). The components were diluted and mixed in excess of *tert*-butanol, followed by lyophilization. Liophylized material was resuspended in PBS, vortex-mixed for 5 min, and sonicated for 15 min before injections. Female athymic nude mice (NCRNU-F) were purchased from Taconic (Rensselaer, NY). Mice were intraperitoneally (IP) injected with A2780CP20 (5x10^5^ cells in 0.2 mL PBS), and seven days later, mice were injected (IP) with 5 µg of either CNT-OMM (N=14) or miR-18a-OMM (N=16) liposomes. Treatments were performed twice per week for three weeks. Two days after the last injection, mice were euthanized, and the number of nodules (primary tumor + nodules), and the tumor weight (primary tumor + nodules) were recorded.

### Bioinformatic Analysis for miR-18a-5p Target Prediction

We used the miRWalk platform to predict the potential miR-18a-5p targets (18). In miRWalk, we marked the comparison analysis box to obtain targets predicted by five different programs: miRDB, miRanda, RNA22, miRWalk, and TargetScan. We selected the miR-18a target genes predicted by at least three of these programs. From this list of genes, we selected the potential genes that were found upregulated (P<0.05 and fold-change > 3) (as miR-18a was reduced) in cisplatin-resistant ovarian cancer relative to their cisplatin-sensitive counterparts in a previously published work (15).

### Quantitative Real-Time PCR (qRT-PCR) to Validate Predicted miR-18a-5p Targets

We designed a 96-well plate from Bio-Rad containing primers to each of the selected miR-18a target genes. Cells (3x10^4^ cells/ml) were plated in 6-well plates, and the next day, cells were transfected with CNT-OMM or miR-18a-OMM, as described above. Twenty-four h later, RNA was isolated with the GenElute Mammalian Total RNA Isolation Kit (Sigma-Aldrich) as per the manufacturer’s instructions. Complementary DNA (cDNA) synthesis was performed with the iScript cDNA synthesis kit (Bio-Rad) using 1 µg of total RNA as starting material. The cDNA was diluted to a concentration of 10 ng/µL to have a final amount of 10 ng of cDNA per well in the designed 96-well plate. SsoAdvanced SYBR Green Supermix (Bio-Rad) was used to perform quantitative PCR on a StepOnePlus instrument. Fold-change and Rq values were calculated relative to CNT-OMM treatment and normalized to β-actin expression.

### Western Blot Analysis

Protein extraction, quantification, and western blot was performed as previously described (19). The membranes were probed with SLC12A6 (Boster Bio, Pleasanton, CA), MMP-3 (Boster Bio), SSX2IP (Novus Biologicals, Centennial, CO), TRAIL-R4 (Boster Bio), GBP1 (Novus), GUCY1A3 (Novus) or TRPC4 (Boster Bio) primary antibodies. Membrane developing was conducted by enhanced chemiluminescence and autoradiography in an SRX-101A film processor (Konika Minolta, Japan). The ImageLab software was used to quantify the density of bands. All membranes were reprobed with a β-actin monoclonal antibody (Sigma) as a loading control.

### Luciferase Reporter Assays

An MMP-3 cDNA clone (that includes the 3’UTR and the firefly luciferase reporter gene) was purchased from OriGene (Cat # SC204366, NM_002422, Rockville, MD) and a renilla vector for luciferase activity normalization was purchased from Promega (pGL4.75[hRluc/CMV], Cat #E6931, Madison, WI). A2780CP20 cells (70,000 cells) were seeded into 6-well tissue culture plates and 24-h later, 3’UTR clones or control vector (1.5 ug) were co-transfected with renilla vector (0.05 µg) using lipofectamine 3000 (ThermoFisher Scientific) on a 1:2.5 (w/v) ratio (DNA:lipofectamine). Six h after plasmid transfection, OMMs (100 nM) were transfected overnight, as described above. The next day, fresh media was added and 48-h after OMM transfection, cells were collected, and luciferase activity was measured with the Dual-Luciferase Reporter Assay System (Promega, Madison, WI) as per manufacturer instructions. Luminescence measurements were conducted on a GLOMAX 20/20 Luminometer (Promega). A two-step normalization was performed with the renilla vector and the 3’UTR vectors. Luciferase activity was calculated relative to CNT-OMM.

### Survival Analysis

Kaplan–Meier survival analysis was performed using publicly available miRNA expression datasets (miRpower for pan-cancer) in Kaplan–Meier (KM) plotter (www.kmplot.com). By selecting the miR-18a gene symbol, hsa-miR-18a, ovarian cancer patients were split into high and low expression groups by the best cut-off values of miRNA expression determined by the best performing threshold between the lower and upper quartile. KM survival plot for overall survival (OS) was generated for all ovarian cancer patients (n=486) without any other restriction (stage, grade, age). P-values <0.05 were considered to be statistically significant.

### Statistical Analysis

Statistical analyses and graphs construction were performed with the GraphPad Prism software (GraphPad Software Inc, La Jolla, CA). P-values were calculated by parametric (t-test or ANOVA) analysis as determined by normality tests. P-values < 0.05 were considered statistically significant.

## Results

### Identification of Differentially Abundant miRNAs in Human Ovarian Cancer Cells

miRNA expression profiles were performed with an Affymetrix array (GeneChip miRNA 2.0) in cisplatin-resistant cell lines (A2780CP20 and A2780CIS) and their cisplatin-sensitive counterpart (A2780). A list of 6,627 miRNAs was initially identified (data not shown). Those miRNAs that were altered by at least 1.5-fold and a P-value of at least 0.05 were considered as potential candidates. With these parameters, we identified 63 miRNAs differentially abundant in A2780CP20 vs. A2780 cells, and 62 miRNAs differentially abundant in A2780CIS vs. A2780 cells (**Supplementary Table S2**). Twenty-six miRNAs were present in both lists (**Table 1**). In this Table, 14 miRNAs were upregulated and 12 miRNAs were downregulated in A2780CP20 and A2780CIS cells compared with A2780 parental cells. Interestingly, several members of the miR-17-92 cluster and its mammalian paralog miR-106a-363 were downregulated. Contrastingly, several members of the let-7 family were upregulated in cisplatin-resistant vs. cisplatin sensitive cells.

**Table 1:** Dysregulated miRNAs in cisplatin-resistant ovarian cancer (CP20 and CIS) vs. cisplatin-sensitive parental A2780.

Transcript ID	P-Value (CIS vs. A2780)	Fold-Change (CIS vs. A2780)	P-Value (CP20 vs. A2780)	Fold-Change (CP20 vs. A2780)
hsa-miR-200c-3p	0.0003	12.6	0.0000	160.4
hsa-let-7b-5p	0.0001	149.7	0.0001	124.7
hsa-miR-132-3p	0.0068	11.7	0.0032	24.7
hsa-let-7d-5p	0.0001	48	0.0001	22.5
hsa-let-7a-5p	0.0000	24.2	0.0000	11.7
hsa-miR-183-5p	0.0024	8.8	0.0029	7.6
hsa-miR-182-5p	0.0161	3.2	0.0044	6.3
hsa-let-7c-5p	0.0008	9.2	0.0020	4.9
hsa-miR-4429	0.0420	1.9	0.0137	2.6
hsa-miR-185-5p	0.0286	2.5	0.0271	2.5
hsa-let-7e-5p	0.0542	1.9	0.0322	2.3
hsa-miR-23b-3p	0.0013	2.9	0.0032	2.2
hsa-miR-4776-3p	0.0112	1.5	0.0064	1.6
hsa-let-7i-5p	0.0006	5.4	0.0321	1.5
hsa-miR-450a-1-3p	0.0103	−1.6	0.0152	−1.5
hsa-miR-4786-3p	0.0060	−1.7	0.0060	−1.7
hsa-miR-619-3p	0.0252	−1.5	0.0087	−1.8
hsa-miR-92a-3p	0.0044	−6.5	0.0499	−2.1
hsa-miR-335-5p	0.0144	−1.7	0.0062	−2.1
hsa-miR-17-5p	0.0000	−6.3	0.0002	−2.7
hsa-miR-106a-5p	0.0009	−7.2	0.0062	−2.7
hsa-miR-18a-5p	0.0000	−8.5	0.0003	−3.1
hsa-miR-20a-5p	0.0005	−7.4	0.0018	−3.8
hsa-miR-92a-1-5p	0.0066	−14.8	0.0380	−4.1
hsa-miR-19b-3p	0.0095	−12.2	0.0105	−11.2
hsa-miR-221-3p	0.0162	−7.4	0.0084	−12.7

**Figure 1:**
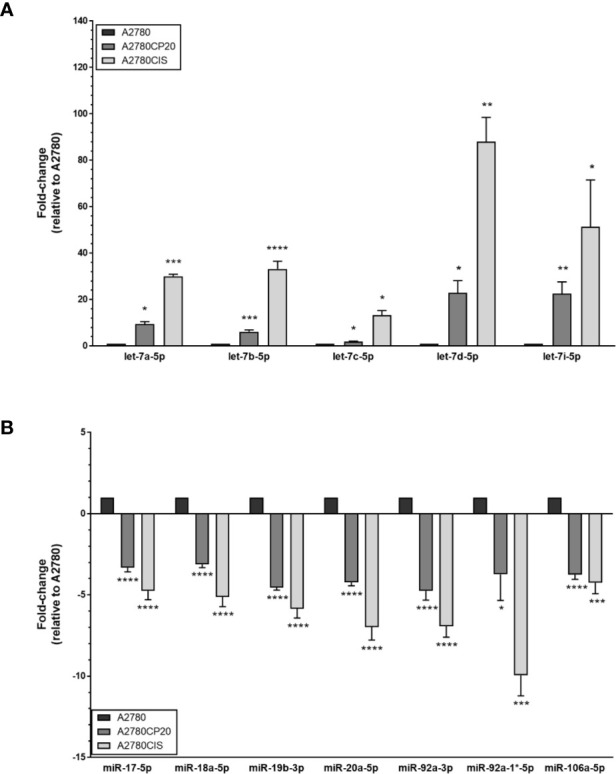
MiRNA expression levels in ovarian cancer cells. RNA was isolated from ovarian cancer cells (A2780CP20, A2780CIS and A2780) followed by TaqMan-based qRT-PCR to validate the microRNA expression array results. **(A)** Upregulated and **(B)** downregulated miRNAs in cisplatin-resistant (A2780CP20 and A2780CIS) vs. cisplatin sensitive cells (A2780CIS). Results are shown as Mean ± SEM of triplicate experiments (*P < 0.05, **P < 0.01, ***P < 0.001, ****P < 0.0001).

Upregulation of miR-17-92 members and downregulation of Let-7 family members have been reported in several cancer types (20–22). Interestingly, in our miRNA profiles, we observed the opposite tendencies. Thus, to further investigate the potential contradictory roles of these miRNAs, we selected the members of these families and the miR-106-a-363 cluster (including miR-17-5p, miR-18a-5p, miR-19b-3p, miR-20a-5p, miR-92a-3p, miR-92a-1*-5p, miR-106a-5p, let-7a-5p, let-7b-5p, let-7c-5p, let-7d-5p, and let-7i-5p) for validation by TaqMan-based quantitative Real-Time PCR (qRT-PCR). The qRT-PCR analysis confirmed the miRNA array results, as shown in **Figures 1A, B**. Again, the expression of several members of the miR-17-92 and the miR-106a-363 clusters, which have been proposed as oncomiRs, were decreased in cisplatin-resistant vs. cisplatin sensitive cells (**Table 1** and **Figures 1A, B**).

### Effect of miRNA OMMs on Cell Proliferation

**Figure 2:**
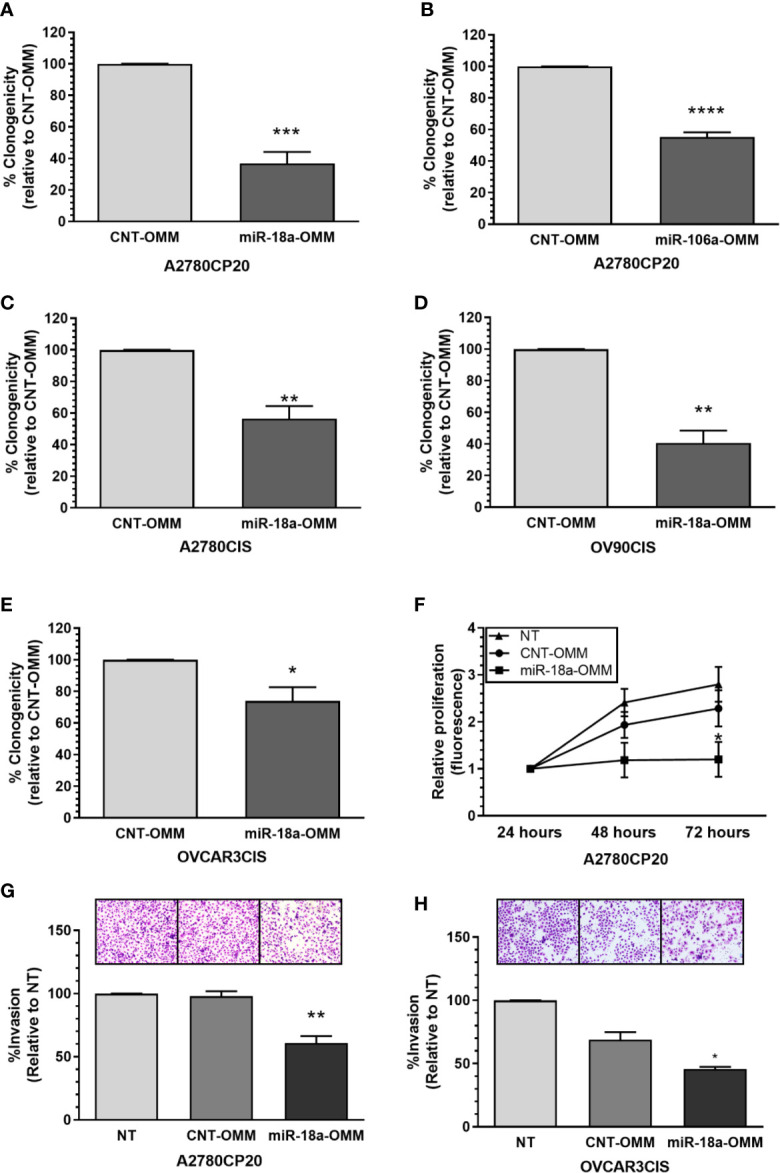
Effect of OMMs in the proliferation and invasion of cisplatin-resistant ovarian cancer cells. Colony formation assay was performed 18 h post-OMM transfection in A2780CP20 cells. **(A)** miR-18a-OMM and **(B)** miR-106-a-OMM. The same trend was observed with **(C)** miR-18a-OMM in A2780CIS cells. Colony formation in **(D)** OV90CIS and **(E)** OVCAR3CIS cells after transfection with miR-18a-OMMs. Percent of clonogenicity was calculated relative to CNT-OMM. **(F)** Cell proliferation was measured by fluorescence using the CyQuant Proliferation kit in A2780CP20 cells 24, 48 and 72 h after transfection with OMMs. Fluorescence was calculated relative to the fluorescence at 24 h post-transfection. Cell invasion was performed after OMMs transfection in **(G)** A2780CP20 cells and **(H)** OVCAR3CIS cells. Untransfected cells were taken as 100% invasion. Experiments were performed in triplicates. All graphs represent mean ± SEM (*P < 0.05, **P < 0.01, ***P < 0.001, ****P < 0.0001).

Since we observed that the miR-17-92 family members showed opposite expression patterns to those reported in the literature, we explored the biological effects of targeting members of this miRNA family using miRNA oligonucleotide mimics (OMM) in a colony formation assay. Compared with a CNT-OMM, transient transfection of miR-18a or miR-106a OMMs in A2780CP20 cells significantly reduced the number of colonies (**Figures 2A, B**, **Supplementary Figure S1**). Particularly, the OMM of miR-18a reduced in more than 70% (***P<0.001) the number of colonies compared with the CNT-OMM in A2780CP20 and more than 40% in A2780CIS (**Figures 2A, C**). Transient transfection of miR-17, miR-92a-1*, miR-19b, miR-20a, and miR-92a OMMs in A2780CP20 cells did not reduce the number of colonies (**Supplementary Figure S2**). Since the greatest effect was observed using the miR-18a-OMM, we focused our studies on the role of this miRNA in the cisplatin resistance of ovarian cancer cells.

Then, we assessed if the miR-18a-OMM inhibited cell proliferation in other ovarian cancer cells, including the HGSOC cell lines OV90CIS and OVCAR3CIS (17). Real-time PCR showed that these cells expressed lower levels of miR-18a as compared with their cisplatin-sensitive counterparts (OV90 and OVCAR3, respectively, **Supplementary Figures S3A, B**). As seen with A2780CP20 and A27870CIS, the miR-18a-OMM reduced cell proliferation in OV90CIS (59% reduction, **P<0.01, **Figure 2D**), and OVCAR3CIS (26% reduction, *P<0.05, **Figure 2E**) cells as compared to the CNT-OMMs. Transient transfection of miR-18a OMMs significantly incremented miR-18a levels in A2780CP20 (***P<0.001), A2780CIS (***P<0.001), OVCAR3CIS (*P<0.05), and OV-90CIS (***P<0.001) as compared with the CNT-OMM (**Supplementary Figures S3C–F**).

### Effect of miR-18a OMM in Cell Proliferation and Invasion

To assess the short-term effect of miR-18a-OMM in the cell proliferation of cisplatin-resistant ovarian cancer cells we performed CyQUANT proliferation assays. Cell proliferation was measured 24, 48, and 72 h post-transfection. Transient transfection of A2780CP20 cells with miR-18a-OMM resulted in gradual decreases of cell proliferation when compared with CNT-OMM. This effect was significant at 72 h post-transfection (47% reduction, *P<0.05, **Figure 2F**). Moreover, we tested whether miR-18a had a role in the invasiveness of A2780CP20 and OVCAR3CIS cells. Upregulation of miR-18a resulted in a significant reduction in the invasion of A2780CP20 (36% reduction, **P<0.01, **Figure 2G**) and OVCAR3CIS cells (23% reduction, *P<0.05, **Figure 2H**) compared to CNT-OMM.

### Therapeutic Effect of Folate-liposomal miR-18a-OMMs

**Figure 3:**
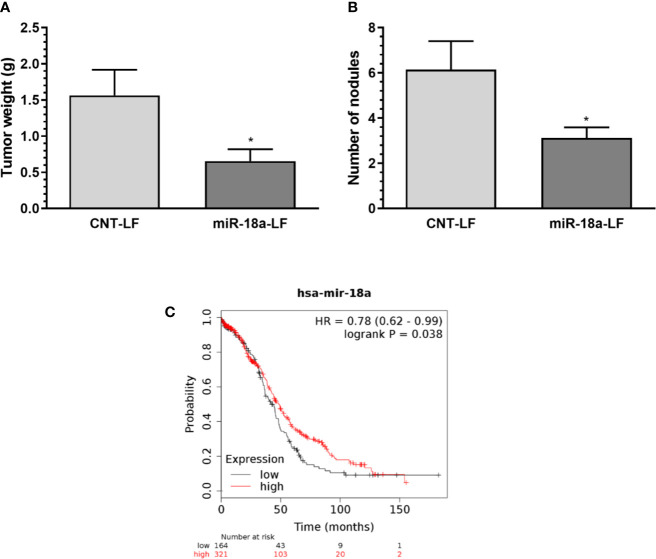
Therapeutic effect of targeting miR-18a *in vivo* and miR-18a clinical relevance. Folate liposomal miR-18a-OMM (miR-18a-LF, N= 16) or CNT-OMM (CNT-LF, N=14) were administered twice a week in intraperitoneal xenograft mouse models of ovarian cancer. **(A)** tumor weight **(B)** number of nodules. Mean ± SEM is shown (*P < 0.05). **(C)** Kaplan-Meier (KM) plot shows that the OS is increased for ovarian cancer patients with higher miR-18a expression levels. Plot was generated using the Kaplan–Meier (KM) plotter (www.kmplot.com).

We then investigated the therapeutic potential of miR-18a-OMM in a xenograft mouse model of cisplatin-resistant ovarian cancer. Evidence indicates that ovarian cancer cells express higher folate receptor alpha (FRα) compared with other cells of the body (23). Based on this information, OMMs were encapsulated in a folate-liposomal formulation. We inoculated (i.p.) A2780CP20 cells into female nude mice and administered folate-liposomal formulations (i.p.) containing miR-18a-OMMs or CNT-OMMs for three weeks twice per week. We observed a significant reduction of tumor weight (*P<0.05) in the miR-18a-OMM treated group compared with the CNT-OMM treated group (**Figure 3A**). In addition, the number of nodules was significantly lower (*P<0.05) in the miR-18a-OMM group in comparison with the CNT-OMM group (**Figure 3B**). These results suggest that folate-targeting liposomes loaded with miR-18a-OMM induced positive therapeutic effects in an ovarian cancer mouse model.

### Expression of miR-18a in Human Ovarian Cancer Patients

To assess the clinical relevance of miR-18a in ovarian cancer, we performed Kaplan-Meier analysis using the KM-plotter online tool. Kaplan-Meier curves (**Figure 3C**) showed that high levels of miR-18a significantly (P<0.0381) increased the Overall Survival (OS) in ovarian cancer patients (41.97 months vs. 48.37 months). These results indicate that miR-18a is associated with the survival time of ovarian cancer patients.

### Identification of miR-18a-5p Target Genes in Cisplatin-Resistant Ovarian Cancer

**Figure 4:**
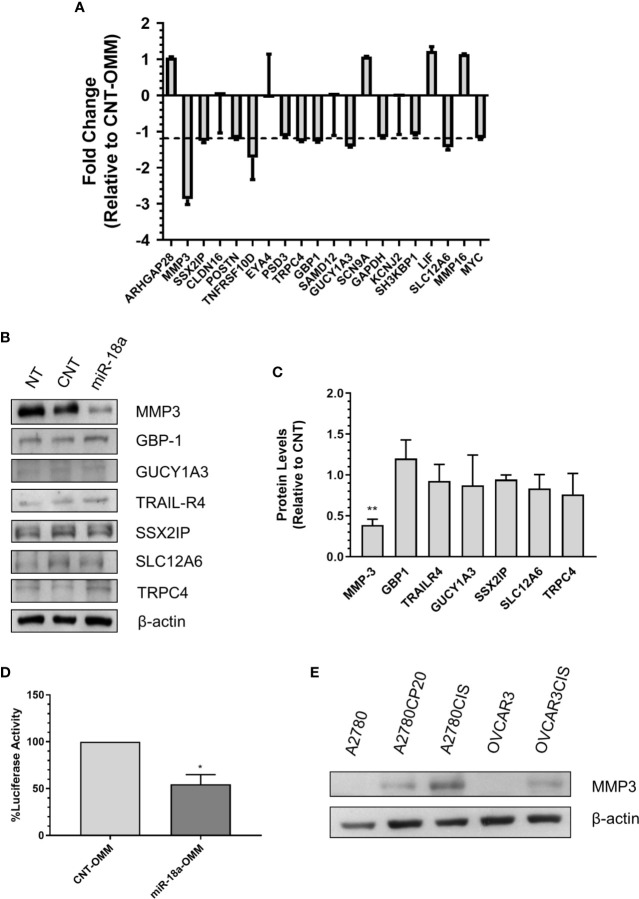
Identification of miR-18a target genes in cisplatin-resistant ovarian cancer. **(A)** qRT-PCR was performed with total RNA extracted from miR-18a-OMM or CNT-OMM transfected A2780CP20 cells. Rq (relative expression) values were calculated relative to CNT-OMM samples. **(B)** Western blot analysis was performed with protein extracts (50 µg) of OMM transfected cells. **(C)** Densitometric analysis of band intensities was performed and relative values were calculated using the intensity of β-actin as control (**P < 0.01). **(D)** Dual**-**luciferase assay was performed to assess the binding of miR-18a to the 3’UTR region of MMP-3. Experiments were performed in triplicates. Mean ± SEM is shown (*P < 0.05). **(E)** Western blot analysis was conducted for MMP-3 with protein extracts of ovarian cancer cell lines.

To further identify the miR-18a-5p downstream effectors in ovarian cancer cells, we investigated the potential miR-18a target genes. An initial bioinformatic analysis using the miRWalk miRNA target prediction comparison tool revealed 1,646 potential miR-18a-targets identified by at least three of the five target prediction programs (data not shown). Since we were interested in targets that may contribute to cisplatin resistance in ovarian cancer, we merged the 1,646 predicted targets with a previously published expression array of cisplatin-resistant and cisplatin-sensitive ovarian cancer cells (15). A total 37 out of 1,646 miR-18a predicted genes were upregulated in cisplatin-resistant relative to cisplatin-sensitive cells (P<0.05, Fold-Change > 3). Based on a literature search, fold-changes, and P-values, the list was reduced to 18 potential miR-18a targets (**Supplementary Table S3**). To confirm experimentally that these genes are real miR-18a targets, we transiently transfected A2780CP20 cells with miR-18a-OMM or CNT-OMM. Real-time PCR with total RNA isolated from the transfected cells revealed seven downregulated genes (fold-change less than -1.2) in miR-18a-OMM cells compared with CNT-OMM cells (**Figure 4A**). Therefore, we selected these genes for further validation by Western blot analysis. Western blots showed that the protein levels of SSX2IP, GBP1, GUCY1A3, SLC12A6, TRPC4, and TRAIL-R4 remained unaltered in miR-18a OMM vs. CNT-OMM (**Figures 4B, C**). However, following the transfection of miR-18a OMM, MMP-3 protein levels were prominently reduced (60%, p<0.05) as compared with CNT-OMM, suggesting MMP-3 as a potential miR-18a target gene. To confirm these findings, we performed dual-Luciferase Reporter Assays, which measures the direct binding of miR-18a to the MMP-3 3’UTR mRNA region. **Supplemenary Figure S4A** shows the potential binding region sequence of miR-18a to MMP-3 as predicted by RNAhybrid (https://bibiserv.cebitec.uni-bielefeld.de/rnahybrid). Transient transfection of miR-18a OMM in A2780CP20 cells reduced the luciferase activity compared to CNT-OMM (P<0.05), indicating that miR-18a binds directly to the 3’UTR region of the MMP-3 mRNA (**Figure 4D**). Together these results suggest that MMP-3 is a direct target of miR-18a in ovarian cancer cells.

### Effect of MMP-3 Silencing on Cell Viability, Proliferation, and Invasion

**Figure 5:**
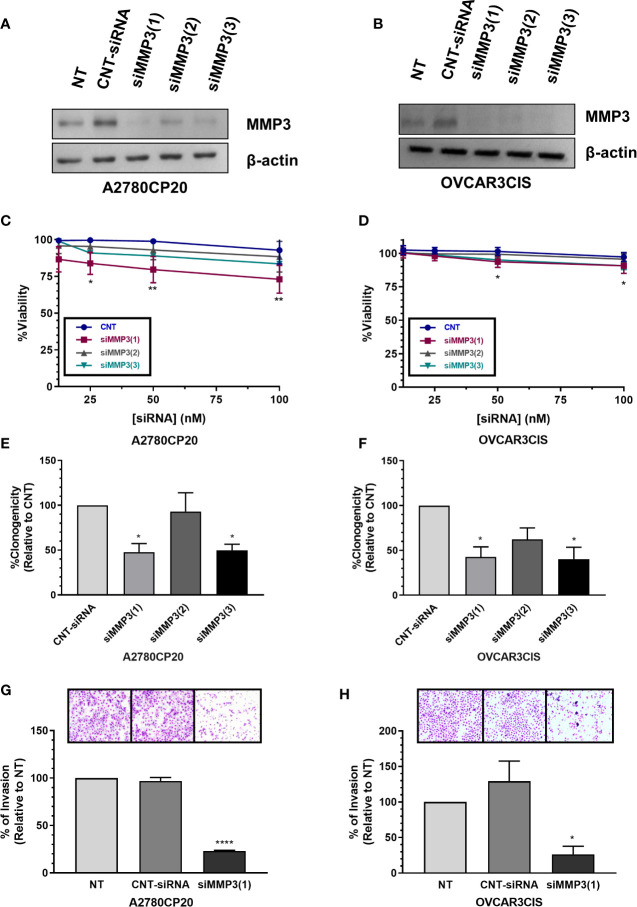
Effect of MMP-3 silencing on cell viability, cell growth and invasion. MMP-3 silencing in **(A)** A2780CP20 and **(B)** OVCAR3CIS was assessed by Western blot analysis of cells collected 24 h after transfection with siRNAs. Cell viability was assessed in **(C)** A2780CP20 and **(D)** OVCAR3CIS with AlamarBlue dye 72 h after siRNA transfection (*P < 0.05, **P < 0.01). Colony formation assay was performed after siRNA transfection in **(E)** A2780CP20 and **(F)** OVCAR3CIS. % of clonogenicity was calculated relative to CNT-siRNA-transfected cells (*P < 0.05). Invasion assay was performed after siRNA (25 nM siRNA, final concentration) transfection in **(G)** A2780CP20 and **(H)** OVCAR3CIS cells. Untransfected cells were taken as 100% invasion. Images of invaded cells were taken with a light microscope on 20X magnification lenses. Mean ± SEM is shown as a result of three independent experiments (*P < 0.05, ****P < 0.0001).

As we found that MMP-3 is a direct target of miR-18a-5p, we further assessed the biological effects of targeting MMP-3 in cisplatin-resistant ovarian cancer. First, we measured the MMP-3 protein levels in a panel of ovarian cancer cells and observed higher levels of this protein in cisplatin-resistant compared with cisplatin-sensitive cells (**Figure 4E**). Next, we assessed the effect of siRNA-mediated MMP-3 silencing on cell viability, cell proliferation, and cell invasion. The Western blots showed in **Figures 5A, B** indicate that transient transfection of MMP-3-targeted siRNAs (siMMP3) in A2780CP20 or OVCAR3CIS cells significantly reduced the MMP-3 protein levels in comparison with a CNT-siRNA. **Supplementary Figures S4B, C** shows the densitometric analysis of the band intensities. A dose-response experiment with MMP-3 siRNAs showed a significant decrease in cell viability at concentrations of 100 (**P<0.01), 50 (**P<0.01) and 25 nM (*P<0.05) of the siMMP3(1) compared to CNT-siRNA (**Figure 5C**). Similar results were observed in the OVCAR3CIS cells (**Figure 5D**). In a colony formation assay, we observed a reduction of about 50% on cell growth in both A2780CP20 and OVCAR3CIS cells transfected with siMMP3(1) (*P<0.05) and siMMP3(3) (*P<0.05) relative to CNT-siRNA transfected cells (**Figures 5E, F**). Transient transfection of the siMMP3(1) reduced the proliferation of A2780CP20 cells at concentrations as low as 25 nM (**Supplementary Figure S4D**). We did not observe any significant changes in cell viability or colony formation assays when cells were transfected with the siMMP3(2). Since the most significant effects were observed with the siRNA(1), we used this siRNA for invasion assays. As it is shown in **Figures 5G, H**, siRNA-mediated MMP-3 targeting significantly reduced the number of invaded cells in A2780CP20 (****P<0.0001) and OVCAR3CIS (*P<0.05) in comparison with the CNT-siRNA.

## Discussion

In this study, we found that miR-18a-5p has a tumor-suppressive role in cisplatin-resistant ovarian cancer cells. The increased levels of miR-18a with OMM decreased *in vitro* cell proliferation and invasion; and the abrogation of tumor growth in a xenograft model of ovarian cancer. Moreover, high levels of miR-18a were associated with a better OS in ovarian cancer patients, suggesting a clinical relevance for this miRNA in this malignancy. Another new finding was that we identified MMP-3 as a novel bonafide target of miR-18a in ovarian cancer cells.

Our miRNA expression profile revealed the dysregulation of several members of the miR-17-92 cluster in cisplatin-resistant vs. cisplatin sensitive cells. The miR-17-92 cluster is one of the best studied miRNA family composed of several polycistronic miRNA genes that code for six different mature miRNAs: MiR-17, miR-18a, miR-19a, miR-20a, miR-19b-1 and miR-92a-1 (20). Members of this cluster have been traditionally associated with oncogenic roles that inhibit apoptosis and promote cell cycle progression (24). Several studies have shown upregulation of the miR-17-92 cluster in different types of cancers, including lung, liver, and thyroid tumors (20, 25, 26). However, we observed reduced expression of various miR-17-92 members in cisplatin-resistant compared with cisplatin-sensitive ovarian cancer cells. In particular, we observed that miR-18a could have a tumor-suppressive role in ovarian cancer as increased expression of this miRNA reduced cell growth, proliferation, invasion, and inhibited tumor growth *in vivo*. These results confirmed previous studies of Liu et al. reporting that miR-18a reduced cell viability and inhibited cell growth in ovarian cancer cells and tumor growth *in vivo* (27). Moreover, Humphreys et al. reported that miR-18a upregulation inhibited cancer progression in gastric and colorectal cancers (28). Contrarily, increased levels of circulating miR-18a were observed in plasma and/or serum of patients with pancreatic, colorectal, esophageal, and hepatocellular cancer compared to healthy patients (29, 30). Similarly, miR-18a-5p (miR-18a) upregulation promoted the progression of nasopharyngeal, hepatocellular and breast carcinoma cell lines (31–33). A possible explanation to these contrasting reports regarding the expression and biological role of miR-18a is that depending on the tumor cell type, miR-18a regulates a particular group of genes, some with oncogenic roles and others with tumor-suppressive capabilities. For example, Huang et al. observed that Smad2, a downstream effector of the TGF-β signaling and a potent tumor suppressor in several cancers, is a major target of miR-18a in oral squamous cell carcinoma (34). The tumor suppressor genes PTEN and IRF2 have also been reported as direct targets of miR-18a in osteosarcoma (35, 36). Another possibility is that while in tumor cells miR-18a could act as a tumor suppressor; in cells of the tumor microenvironment, miR-18a could have an oncogenic role. For instance, Mitra et al. found downregulation of miR-214 in cancer-associated fibroblasts (CAF), contrasting to the oncogenic role and upregulation of this miRNA in tumor cells (37). Therefore, the precise expression patterns of miR-18a in ovarian tumor and its tumor microenvironment should be further investigated.

Regarding the miR-18a downstream effectors, Liu et al. identified TRIAP1 and IPMK as direct targets of miR-18a in epithelial ovarian cancer cells (27). Our approach to identify the miR-18a targets included bioinformatic analyses followed by qPCR, Western blots, and dual-luciferase reporter assays. Starting with several potential target genes, we confirmed only MMP-3 as a direct miR-18a target in ovarian cancer cells. MMP-3, also known as stromelysin-1, is part of the family of proteins categorized as matrix metalloproteinases (MMPs). MMPs are involved in the degradation of components of the extracellular matrix (ECM) and therefore play a significant role in processes involving cellular remodeling such as cell migration, invasion and epithelial-to-mesenchymal transition (EMT) (38). Moreover, MMPs levels are elevated on a large number of malignancies, and high expression of MMP has been correlated with poor prognosis in several cancers (39). Due to their central role as regulators of the ECM structure, a required step for cancer cell migration and metastasis, MMP inhibitors were widely considered as targets for cancer therapy even in clinical trials (40). However, these studies were abandoned due to the overt toxicities resulting from the non-specific profile of these synthetic and natural MMP inhibitors (41). Here, we showed that siRNA-mediated MMP-3 silencing reduced cell viability, cell growth, and invasion of cisplatin-resistant ovarian cancer. These findings open a new therapeutic opportunity using siRNA-related approaches, avoiding in this way the limitations observed with the MMPs small inhibitors. Nonetheless, therapeutic experiments are needed before proposing MMP-3 as a therapeutic target for ovarian cancer treatment.

Although MMP-3 has been studied in several cancers (42, 43), there are limited reports of the role of MMP-3 in ovarian cancer. However, using RNA-Seq and TCGA data, researchers found MMP-3 overexpression in high-stage and high-grade ovarian tumors compared to lower stages tumors (44). In this study, we explored the biological function of MMP-3 in cisplatin-resistant ovarian cancer. Nevertheless, critical questions remain unanswered. For example, what is the molecular mechanism connecting MMP-3, a protein of the ECM, with intracellular proteins involved in cisplatin resistance. In this regard, Bissell and co-workers performed a proteomic screening of MMP-3-binding partners in mouse mammary epithelial cells and found that the intracellular chaperone heat-shock protein 90β (HSP90β) interacts extracellularly with the hemopexin domain of MMP-3 (45). This interaction was necessary for the invasion of mammary epithelial cells. Likewise, Werb and co-workers reported that MMP-3 regulates the Wnt pathway by interacting with the ligand Wnt5b in the mammary stem cell activity (46). Also, Si-Tayeb et al. and Eguchi et al. observed MMP-3 in the nucleus of cells (47, 48). This portion of MMP-3 could promote the activation of genes involved in the drug resistance of ovarian cancer cells. Besides the posttranscriptional regulation of MMP-3 by miR-18a, transcriptional regulation is possible. For instance, in lung cancer, IL-6 regulates the expression of MMP-3 through ATM phosphorylation, a potential factor associated with drug resistance in this cancer type (49). These hypotheses should be addressed in the future.

The transcriptional regulation of the miR-17-92 cluster occurs mainly by the c-MYC oncogene. N-MYC and the Notch signaling pathway may also transcriptionally regulate the expression of the miR-17-92 cluster (50, 51). However, c-MYC is also increased in cisplatin-resistant ovarian cancer cells (19), which would result in the upregulation of the miR-17-92 cluster, including miR-18a. Therefore, mechanisms inhibiting the expression of the miR-17-92, even at the posttranscriptional level, must occur in cisplatin-resistant ovarian cancer cells. For example, there has been reports of long non-coding RNAs (lncRNA) counteracting the oncogenic action of the miR-17-92 cluster (52, 53). In retinoblastoma, for instance, the lncRNA H19 binds on seven regions to the miR-17-92 cluster members miR-17, miR-18a, miR-19a, miR-19b, and miR-20a suppressing their activity, and inhibiting cancer progression (53). Additionally, genome-wide analysis has shown deletion of this cluster in almost 17% of ovarian tumors (54). The molecular mechanisms leading to miR-18a downregulation in cisplatin-resistant ovarian cancer cells should be further investigated.

## Conclusions

This study provides evidence that cisplatin-resistant ovarian cancer cells express lower levels of miR-18a compared with their cisplatin-sensitive counterparts. Targeting miR-18a with OMM-loaded folate-liposomes has therapeutic potential for women diagnosed with cisplatin-resistant ovarian cancer. As MMP-3 is a direct miR-18a-regulated gene in ovarian cancer, MMP-3 is also a promising target for siRNA-based therapies in ovarian cancer and other malignancies.

## Data Availability Statement

The original contributions presented in the study are publicly available. This data can be found here: https://www.ncbi.nlm.nih.gov/geo/, accession no. GSE161784.

## Ethics Statement

The animal study was reviewed and approved by University of Puerto Rico Institutional Animal Care and Use Committee (IACUC).

## Author Contributions

PEVM conceptualized and supervised the project. BIQD, JMRG, VSG, ICD, FV, and GSSS performed the experiments and analyzed data. JMRG and PEVM contributed to the editing of the manuscript. BIQD wrote the original draft. All authors contributed to the article and approved the submitted version.

## Funding

This research was funded by RCMI grant U54 MD007600 (National Institute on Minority Health and Health Disparities) from the National Institutes of Health, institutional seed funds from the University of Puerto Rico Comprehensive Cancer Center (PEVM), and the NIGMS-RISE Grant Number R25-GM061838 (BIQD, GSSS, JMRG).

## Conflict of Interest

The authors declare that the research was conducted in the absence of any commercial or financial relationships that could be construed as a potential conflict of interest.
