# Supplementary material for: MicroRNA-18a-5p Suppresses Tumor Growth via Targeting Matrix Metalloproteinase-3 in Cisplatin-Resistant Ovarian Cancer
Source: Front Oncol. 2020 Dec 17;10:602670. doi: 10.3389/fonc.2020.602670 (PMC7774672; doi:10.3389/fonc.2020.602670)
Supplement: Supplementary file 1 [file DataSheet_1.docx]

Supplementary Material

# Supplementary Tables

**Supplementary Table S1**. Sequences of mature miRNAs and siRNAs.

| **miRNA/siRNA** | **Sequence** |
| --- | --- |
| hsa-miR-18a-5p | (5’-TAAGGTGCATCTAGTGCAGATAG-3’) |
| hsa-miR-17-5p | (5’-CAAAGTGCTTACAGTGCAGGTAG-3’) |
| hsa-miR-19b-3p | (5’-TGTGCAAATCCATGCAAAACTGA-3’) |
| hsa-miR-20a-5p | (5’-TAAAGTGCTTATAGTGCAGGTAG-3’) |
| hsa-miR-92a-3p | (5’-TATTGCACTTGTCCCGGCCTGT-3’) |
| hsa-miR-92a-1*-5p | (5’-AGGTTGGGATCGGTTGCAATGCT-3’) |
| hsa-miR-106a-5p | (5’-AAAAGTGCTTACAGTGCAGGTAG-3’) |
| siMMP3(1) | (5’-CACAAUAUGGGCACUUUAA-3’) |
| siMMP3(2) | (5’-GAACAAUGGACAAAGGAUA-3’) |
| siMMP3(3) | (5’-CAUAUGAAGUUACUAGCAA-3’) |

**Supplementary Table S2**. Dysregulated miRNAs on cisplatin-resistant ovarian cancer cells.

| **Transcript ID** | **p-value** | **Fold-Change** |
| --- | --- | --- |
| *A2780CP20 vs. A2780* | | |
| hsa-miR-200c-3p | 0.0000 | 160.4 |
| hsa-let-7b-5p | 0.0001 | 124.7 |
| hsa-miR-132-3p | 0.0032 | 24.7 |
| hsa-let-7d-5p | 0.0001 | 22.5 |
| hsa-let-7a-5p | 0.0000 | 11.7 |
| hsa-miR-183-5p | 0.0029 | 7.6 |
| hsa-miR-182-5p | 0.0044 | 6.3 |
| hsa-miR-1301-3p | 0.0420 | 5.2 |
| hsa-let-7c-5p | 0.0020 | 4.9 |
| hsa-miR-4492 | 0.0109 | 3.4 |
| hsa-miR-3960 | 0.0306 | 2.8 |
| hsa-miR-320b | 0.0075 | 2.7 |
| hsa-miR-4530 | 0.0387 | 2.7 |
| hsa-miR-4429 | 0.0137 | 2.6 |
| hsa-miR-185-5p | 0.0271 | 2.5 |
| hsa-miR-320a | 0.0128 | 2.4 |
| hsa-miR-320c | 0.0219 | 2.4 |
| hsa-let-7e-5p | 0.0322 | 2.3 |
| hsa-miR-23b-3p | 0.0032 | 2.2 |
| hsa-miR-212-3p | 0.0212 | 2.2 |
| hsa-miR-4505 | 0.0340 | 2.1 |
| hsa-miR-6760-3p | 0.0146 | 1.8 |
| hsa-miR-584-3p | 0.0276 | 1.7 |
| hsa-miR-7109-5p | 0.0539 | 1.7 |
| hsa-miR-2467-3p | 0.0044 | 1.6 |
| hsa-miR-4417 | 0.0294 | 1.6 |
| hsa-mir-132 | 0.0008 | 1.6 |
| hsa-miR-4776-3p | 0.0064 | 1.6 |
| hsa-miR-4750-3p | 0.0249 | 1.6 |
| hsa-let-7i-5p | 0.0321 | 1.5 |
| hsa-mir-452 | 0.0346 | 1.5 |
| hsa-miR-5697 | 0.0536 | 1.5 |
| hsa-miR-3687 | 0.0407 | 1.5 |
| hsa-miR-5585-3p | 0.0148 | 1.5 |
| hsa-miR-1249 | 0.0443 | 1.5 |
| hsa-mir-4431 | 0.0409 | 1.5 |
| hsa-miR-6840-3p | 0.0265 | 1.5 |
| hsa-miR-450a-1-3p | 0.0152 | -1.5 |
| hsa-mir-302a | 0.0472 | -1.5 |
| hsa-mir-214 | 0.0073 | -1.6 |
| hsa-miR-6884-5p | 0.0335 | -1.6 |
| hsa-miR-6865-3p | 0.0416 | -1.6 |
| hsa-miR-151a-5p | 0.0216 | -1.6 |
| hsa-miR-5009-5p | 0.0338 | -1.6 |
| hsa-miR-4786-3p | 0.0060 | -1.7 |
| hsa-miR-619-3p | 0.0087 | -1.8 |
| hsa-miR-23a-3p | 0.0383 | -2.0 |
| hsa-miR-125a-5p | 0.0317 | -2.1 |
| hsa-miR-16-5p | 0.0477 | -2.1 |
| hsa-miR-335-5p | 0.0062 | -2.1 |
| hsa-miR-92a-3p | 0.0499 | -2.1 |
| hsa-miR-99a-5p | 0.0272 | -2.2 |
| hsa-miR-26a-5p | 0.0058 | -2.6 |
| hsa-miR-106a-5p | 0.0062 | -2.7 |
| hsa-miR-17-5p | 0.0002 | -2.7 |
| hsa-miR-18a-5p | 0.0003 | -3.1 |
| hsa-miR-20a-5p | 0.0018 | -3.8 |
| hsa-miR-92a-1-5p | 0.0380 | -4.1 |
| hsa-miR-199a-5p | 0.0008 | -5.1 |
| hsa-miR-199a-3p | 0.0001 | -6.3 |
| hsa-miR-199b-3p | 0.0001 | -6.3 |
| hsa-miR-19b-3p | 0.0105 | -11.2 |
| hsa-miR-221-3p | 0.0084 | -12.7 |
| *A2780CIS vs. A2780* | | |
| hsa-let-7b-5p | 0.0001 | 149.7 |
| hsa-let-7d-5p | 0.0001 | 48.0 |
| hsa-let-7a-5p | 0.0000 | 24.2 |
| hsa-miR-200c-3p | 0.0003 | 12.6 |
| hsa-miR-132-3p | 0.0068 | 11.7 |
| hsa-let-7c-5p | 0.0008 | 9.2 |
| hsa-miR-183-5p | 0.0024 | 8.8 |
| hsa-miR-27b-5p | 0.0172 | 5.9 |
| hsa-let-7f-5p | 0.0344 | 5.5 |
| hsa-let-7i-5p | 0.0006 | 5.4 |
| hsa-miR-27b-3p | 0.0160 | 4.0 |
| hsa-miR-196a-5p | 0.0119 | 3.3 |
| hsa-miR-182-5p | 0.0161 | 3.2 |
| hsa-miR-23b-3p | 0.0013 | 2.9 |
| hsa-let-7g-5p | 0.0479 | 2.8 |
| hsa-miR-324-5p | 0.0325 | 2.5 |
| hsa-miR-185-5p | 0.0286 | 2.5 |
| hsa-miR-99a-5p | 0.0244 | 2.2 |
| hsa-miR-194-5p | 0.0099 | 2.0 |
| hsa-let-7e-5p | 0.0542 | 1.9 |
| hsa-miR-4429 | 0.0420 | 1.9 |
| hsa-miR-574-5p | 0.0107 | 1.9 |
| hsa-miR-24-3p | 0.0263 | 1.8 |
| hsa-miR-3198 | 0.0208 | 1.7 |
| hsa-mir-365a | 0.0472 | 1.6 |
| hsa-miR-4433-3p | 0.0321 | 1.6 |
| hsa-miR-193a-3p | 0.0020 | 1.6 |
| hsa-mir-365a | 0.0458 | 1.6 |
| hsa-miR-4329 | 0.0528 | 1.5 |
| hsa-miR-4279 | 0.0195 | 1.5 |
| hsa-miR-4776-3p | 0.0112 | 1.5 |
| hsa-miR-620 | 0.0252 | 1.5 |
| hsa-miR-518f-3p | 0.0429 | 1.5 |
| hsa-miR-495-3p | 0.0051 | -1.5 |
| hsa-miR-6782-5p | 0.0417 | -1.5 |
| hsa-miR-553 | 0.0131 | -1.5 |
| hsa-miR-3194-3p | 0.0365 | -1.5 |
| hsa-mir-1247 | 0.0182 | -1.5 |
| hsa-miR-6069 | 0.0091 | -1.5 |
| hsa-mir-17 | 0.0458 | -1.5 |
| hsa-mir-519e | 0.0504 | -1.5 |
| hsa-miR-619-3p | 0.0252 | -1.5 |
| hsa-miR-5581-3p | 0.0165 | -1.5 |
| hsa-mir-548a-1 | 0.0117 | -1.5 |
| hsa-mir-361 | 0.0002 | -1.6 |
| hsa-miR-450a-1-3p | 0.0103 | -1.6 |
| hsa-mir-5188 | 0.0396 | -1.6 |
| hsa-miR-4284 | 0.0375 | -1.6 |
| hsa-mir-451b | 0.0051 | -1.6 |
| hsa-miR-4786-3p | 0.0060 | -1.7 |
| hsa-miR-4722-3p | 0.0350 | -1.7 |
| hsa-miR-335-5p | 0.0144 | -1.7 |
| hsa-miR-1587 | 0.0390 | -1.9 |
| hsa-miR-361-5p | 0.0012 | -2.5 |
| hsa-miR-17-5p | 0.0000 | -6.3 |
| hsa-miR-92a-3p | 0.0044 | -6.5 |
| hsa-miR-106a-5p | 0.0009 | -7.2 |
| hsa-miR-20a-5p | 0.0005 | -7.4 |
| hsa-miR-221-3p | 0.0162 | -7.4 |
| hsa-miR-18a-5p | 0.0000 | -8.5 |
| hsa-miR-19b-3p | 0.0095 | -12.2 |
| hsa-miR-92a-1-5p | 0.0066 | -14.8 |

**Supplementary Table S3**. Selected miRNA-18a potential target genes as identified by bioinformatic analysis

| **Gene Symbol** | **Full Name** | **Target Prediction** |
| --- | --- | --- |
| PSD3 | Pleckstrin And Sec7 Domain Containing 3 | miRanda, miRDB, miRWalk, Targetscan |
| CLDN16 | Claudin 16 | miRanda, miRWalk, Targetscan |
| EYA4 | EYA Transcriptional Coactivator And Phosphatase 4 | miRanda, miRWalk, Targetscan |
| SCN9A | sodium voltage-gated channel alpha subunit 9 | miRanda, miRWalk, Targetscan |
| ARHGAP28 | Rho GTPase Activating Protein 28 | miRanda, miRWalk, Targetscan |
| MMP3 | matrix metallopeptidase 3 | miRanda, miRWalk, RNA22, Targetscan |
| KCNJ2 | Potassium Voltage-Gated Channel Subfamily J Member 2 | miRanda, miRDB, miRWalk, Targetscan |
| TRPC4 | transient receptor potential cation channel subfamily C member 4 | miRanda, miRWalk, Targetscan |
| GBP1 | Guanylate Binding Protein 1 | miRanda, miRWalk, Targetscan |
| SLC12A6 | solute carrier family 12 member 6 | miRanda, miRWalk, Targetscan |
| POSTN | Periostin | miRanda, miRWalk, Targetscan |
| MMP16 | matrix metallopeptidase 16 | miRanda, miRWalk, RNA22, Targetscan |
| GUCY1A3 | Guanylate Cyclase 1 Soluble Subunit Alpha | miRanda, miRWalk, Targetscan |
| SSX2IP | SSX Family Member 2 Interacting Protein | miRanda, miRWalk, Targetscan |
| TNFRSF10D | TNF Receptor Superfamily Member 10d | miRanda, miRWalk, Targetscan |
| SAMD12 | Sterile Alpha Motif Domain Containing 12 | miRanda, miRWalk, RNA22, Targetscan |
| LIF | Interleukin 6 Family Cytokine | miRanda, miRWalk, RNA22, Targetscan |
| SH3KBP1 | SH3 Domain Containing Kinase Binding Protein 1 | miRanda, miRDB, miRWalk, Targetscan |

# Supplementary Figures


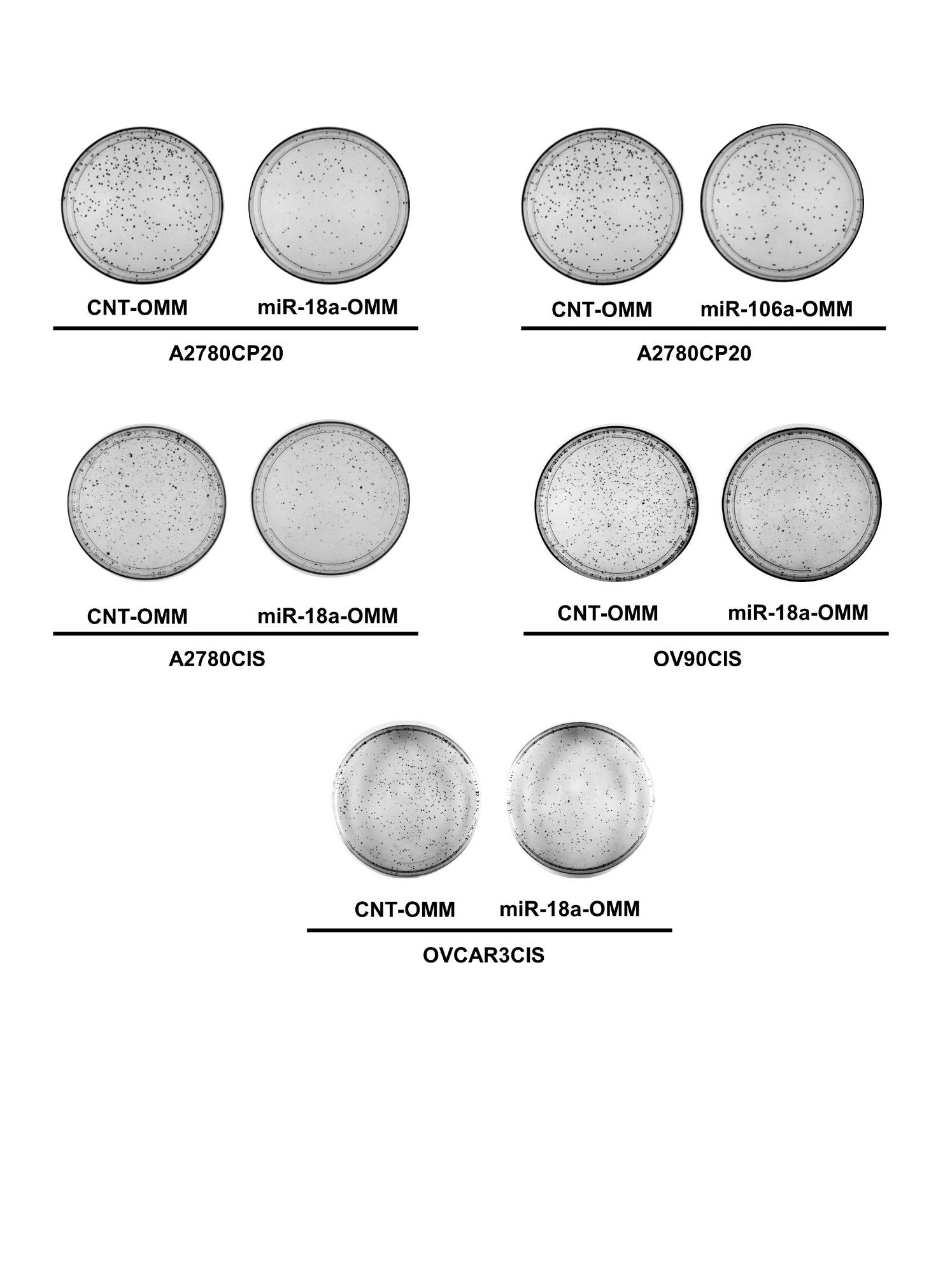


**Supplementary Figure S1.** Representative colony formation assays images described in Figure 1A-E.


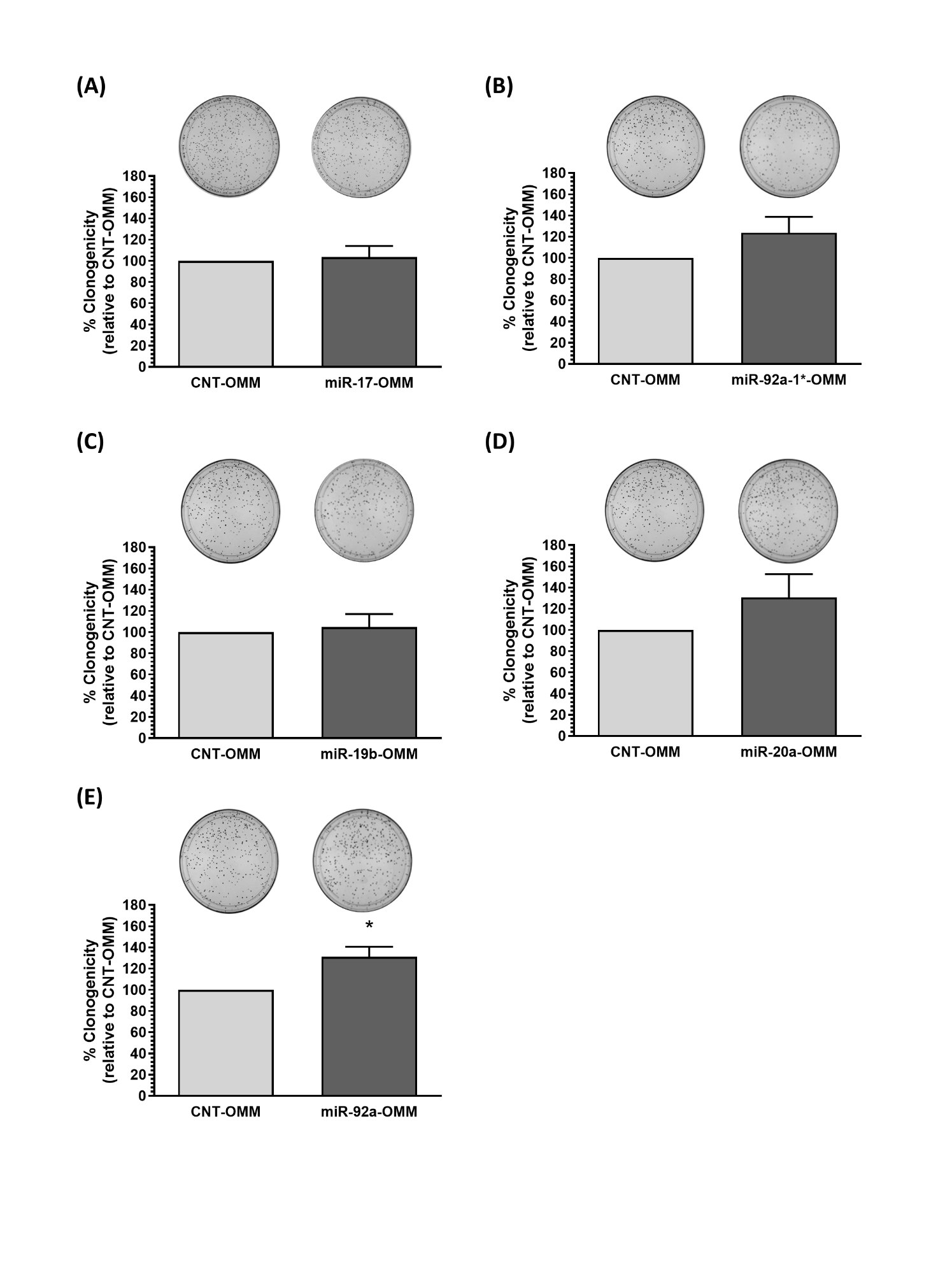


**Supplementary Figure S2.** Colony formation assays performed in A2780CP20 after OMMs transfection as described in “Materials and Methods” (P<0.05).


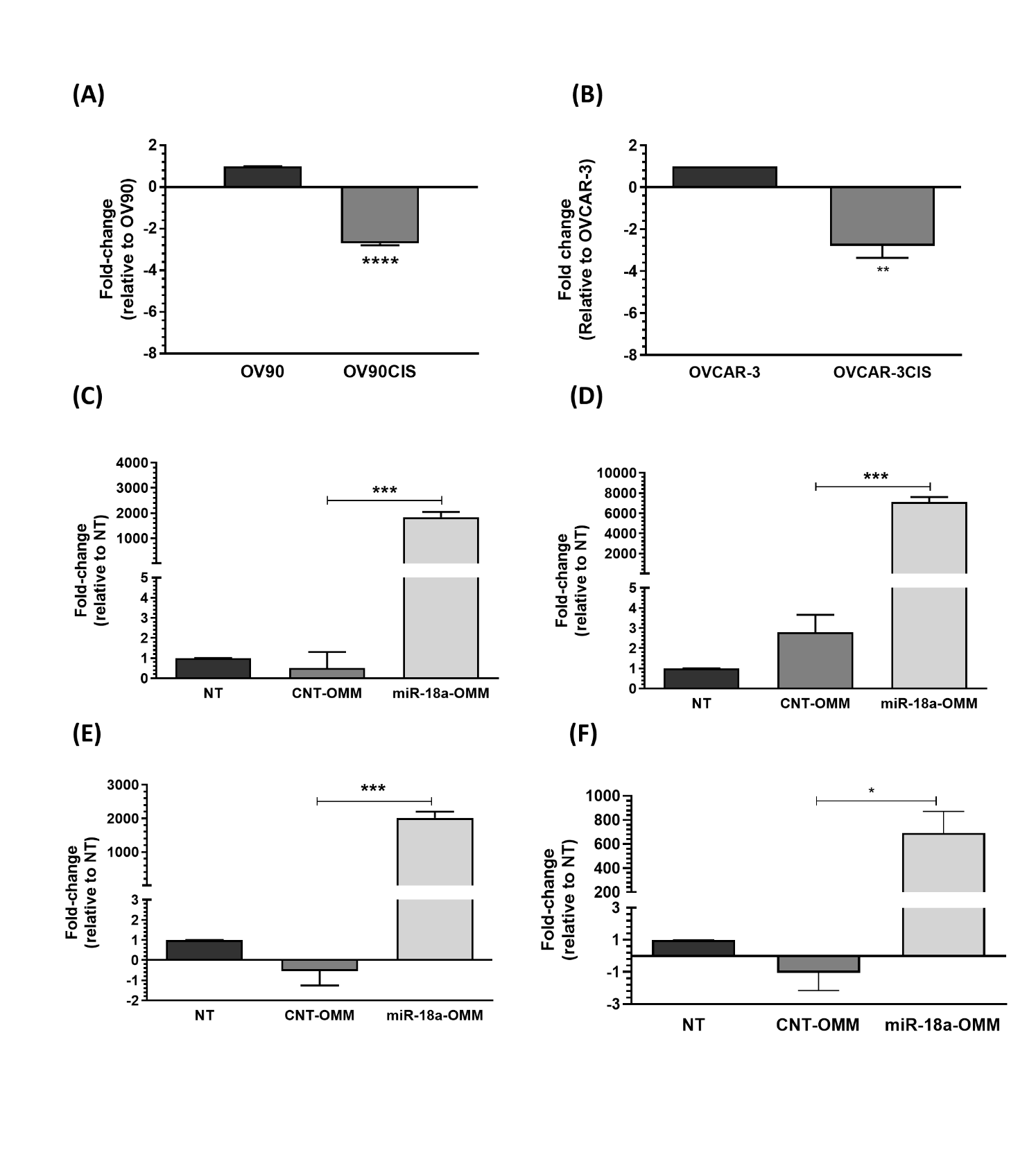


**Supplementary Figure S3.** MicroRNA-18a levels were measured on pairs of cisplatin-sensitive and cisplatin-resistant HGSOC cells, **(A)** OV-90/OV-90CIS and **(B)** OVCAR-3/OVCAR-3CIS**.** qPCR analysis to quantify the miR-18a levels following OMMs transfection in **(C)** A2780CP20, **(D)** A2780CIS, **(E)** OV90CIS and **(F)** OVCAR3CIS. Fold-Changes were calculated relative to non-treated cells. Experiments were performed in triplicates Columns: mean ± SEM (*P<0.05, **<0.01, ***P<0.001).


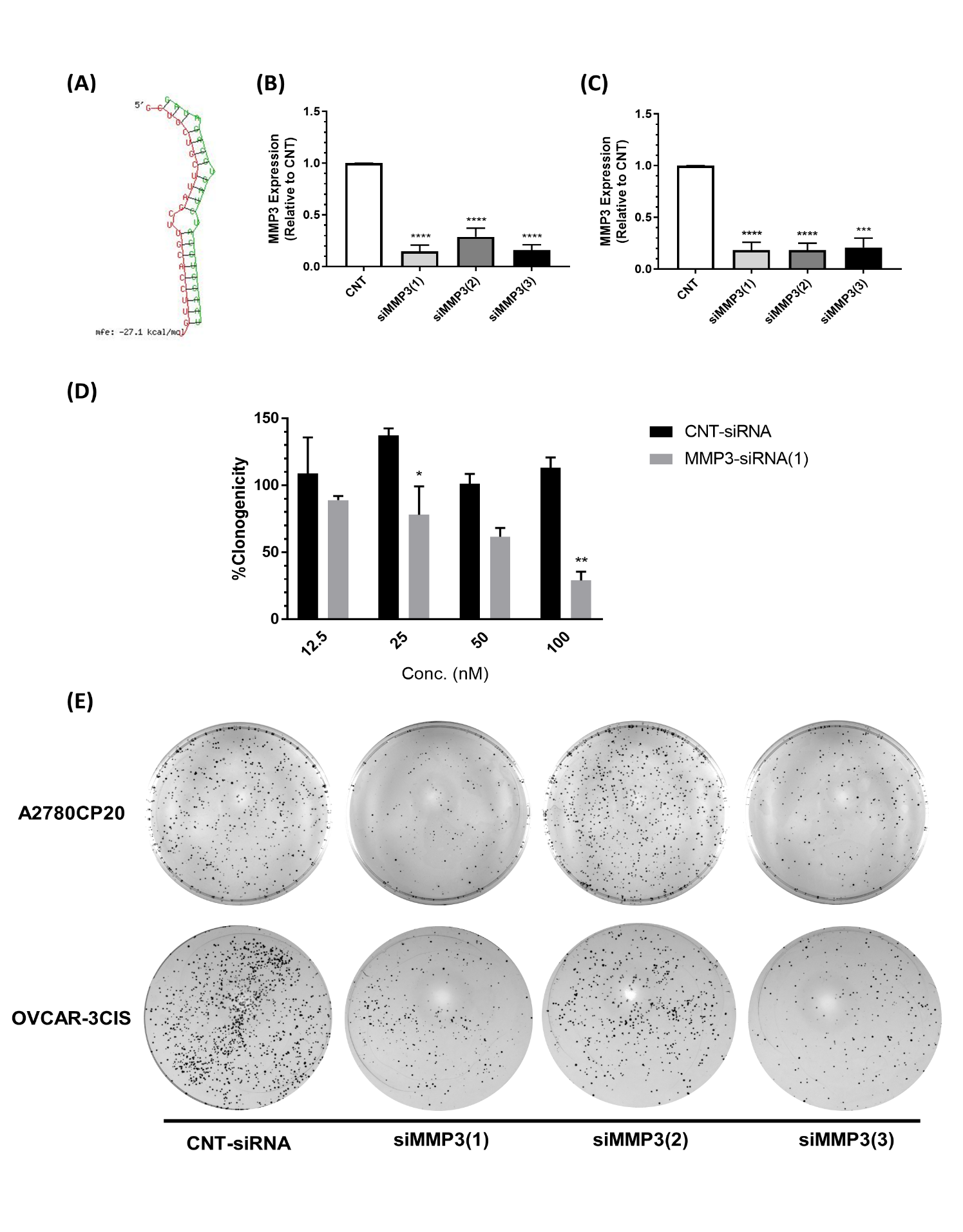


**Supplementary Figure S4. (A)** Potential binding site of miR-18a on the 3’UTR of MMP-3 as predicted by the RNAHybrid program. **(B-C)** Densitometric analysis of Western blot band intensity from Figure 5A-B images showed the downregulation of MMP-3 upon transfection with the three siRNAs. β-actin was used as a housekeeper for normalization. Relative expression was calculated relative to CNT-siRNA samples. Columns: mean ± SEM (**P<0.01, ***P<0.001). **(D)** Colony formation assay in A2780CP20 cells after transfection with 12.5, 25, 50 and 100 nM (final concentration) siMMP3(1). % of clonogenicity was calculated relative to NT (*P<0.05, **P<0.01). **(E)** Representative images of colony formation assays from Figure 5E-F. Experiments on C and D were performed in triplicates.
